# Association between fish consumption and mortality in the E3N French women’s cohort

**DOI:** 10.3389/fnut.2024.1462710

**Published:** 2024-10-30

**Authors:** Cira Ba, Chloé Marques, Pauline Frenoy, Xuan Ren, Gianluca Severi, Francesca Romana Mancini

**Affiliations:** ^1^Université Paris-Saclay, UVSQ, Inserm, Gustave Roussy, CESP, Villejuif, France; ^2^Department of Statistics, Computer Science, Applications “G. Parenti”, University of Florence, Florence, Italy

**Keywords:** fish, omega 3, mortality, persistent organic pollutant, women, cohort

## Abstract

Western studies have shown a non-linear association between fish consumption and mortality, which might be explained by exposure to chemical contaminants. This study aims to explore the associations between fish consumption or omega-3 polyunsaturated fatty acids (n-3 PUFA) and mortality within the prospective E3N French cohort, and to investigate the role of dietary exposure to contaminants in these associations. In the E3N cohort composed of 72,585 women, we assessed fish consumption and n-3 PUFA intake through a food questionnaire sent in 1993. To estimate the dietary exposure to contaminants, we used the food contamination database of the second French total diet study. Cox proportional hazard models were used to estimate the association between fish, lean fish, fatty fish, and n-3 PUFA intake, with the risk of all-cause or cause-specific mortality. During the follow-up (1993–2014), 6,441 deaths were recorded. A U-shaped association was observed between fish consumption and all-cause mortality (P_overall_association_ = 0.017). A similar association was observed with lean fish consumption, while the non-linear association between fatty fish consumption or n-3 PUFA intake and all-cause mortality did not reach statistical significance. A non-linear association was observed between fish consumption and lung cancer mortality (P_overall_association_ = 0.005). A positive and linear association was observed between fatty fish consumption or n-3 PUFA intake and breast cancer mortality (HR [CI95%]: 1.07 [1.01–1.15] and 1.08 [1.01–1.15]). Our results remained unchanged when further adjusting on dietary exposure to contaminants. Our results showed a U-shaped association between fish consumption and all-cause mortality and suggest a notable role of lean fish consumption in this association, but no role of dietary exposure to contaminants. Further studies are needed to better clarify this U-shaped association and the different impacts of fatty and lean fish consumption on health.

## 1 Introduction

Fish has valuable nutritional qualities that make it a particularly interesting food from a nutritional point of view. The European Food Safety Authority (EFSA) recommends eating fish twice a week, including fatty fish (1). Fish is a source of proteins and essential micronutrients and constitutes a primary source of long-chain omega-3 polyunsaturated fatty acids (n-3 PUFA): eicosapentaenoic acid (EPA), docosapentaenoic acid (DPA) and docosahexaenoic acid (DHA) (2). A recent meta-analysis showed that n-3 PUFA were associated with a lower risk of developing major chronic diseases, including cardiovascular disease (CVD), coronary heart disease (CHD), and overall mortality (3). Previous epidemiological studies that have investigated the association between fish consumption and mortality risk generally indicate that consuming fish reduces the risk of mortality (4–6). However, findings from epidemiological studies remain heterogeneous. Several meta-analyses have identified a linear inverse or non-linear U-shaped association between fish consumption and the risk of all-cause mortality (3, 7), with variations observed based on geographical regions. Indeed, whereas some Asian studies showed a linear, inverse, and statistically significant association (6, 8), Western studies showed a non-linear U-shaped association that was not statistically significant (4, 5). It has been suggested that the shape of the association between fish consumption and mortality risk may depend on several factors, such as fish preparation methods (fried or not) (7), fat content (9, 10), or level of contamination from chemical substances (11, 12). Indeed, increased fish consumption may be linked to elevated risks of all-cause and cause-specific mortality, due to heightened exposure to chemical contaminants, such as heavy metals (methylmercury (MeHg), cadmium) and persistent organic pollutants (POPs; such as dioxins, polychlorobiphenyls (PCBs), polybrominated diphenyl ethers (PBDEs) (11–13). Exposure to environmental contaminants in fish might reduce the positive impacts of fish consumption and potentially elucidate the U-shaped associations observed in prior studies on all-cause mortality.

The present study aims to explore the association between fish consumption and all-cause and cause-specific mortality, along with the association between the intake of n-3 PUFA (EPA, DHA, and DPA) and all-cause and cause-specific mortality within the French E3N (Epidemiological Study on Women of the National Education) prospective cohort. As a secondary objective, this study will estimate the direct effect of fish on mortality by adjusting on dietary exposure to food chemical contaminants, namely POPs.

## 2 Materials and methods

### 2.1 E3N cohort

The E3N study launched in 1990 across mainland France is an ongoing prospective cohort that included 98,995 women aged from 40 to 65 years at enrolment, living in France, and insured by MGEN, a health insurance for workers of the French national education system. Anthropometric, lifestyle, and health characteristics were systematically gathered every 2 to 3 years through self-administered questionnaires. Only 3% of E3N women were lost to follow-up (14). All participants provided explicit consent for their involvement in this study, which received approval from the CNIL (the French National Commission for Data Protection and Privacy).

### 2.2 Ascertainment of mortality

Information regarding the vital status of participants was obtained from various databases, including health insurance records, postal services, municipal registries, physicians, and next of kin. Details about the causes of death were sourced from Inserm-CépiDC (French Epidemiology Center on the Medical Causes of Death). Causes of death were coded according to the ninth revision (ICD-9, death before 2000) and the tenth revision (ICD-10, death after 2000) of the International Classification of Diseases (ICD). Mortality by cause was defined as follows: ICD-9390–459 and ICD-10 I00-I99 for CVD; ICD-9140-208 and ICD-10 C00-C97 for cancer; ICD-9174 and ICD-10 C50 for breast cancer; ICD-9153, 154.0, 154.1 and ICD-10 C18-C20 for colorectal cancer; ICD-9162 and ICD-10 C33-C34 for lung cancer.

### 2.3 Dietary questionnaire

In the E3N cohort, dietary data were collected using a semi-quantitative food frequency questionnaire. This questionnaire comprising 208 food items was distributed in 1993 (third questionnaire, Q3). It consisted of two parts. The first part addressed the frequency of consumption (never, 1 to 3 times per month, or 1 to 7 times per week) and the quantity consumed of various food groups during 8 meal occasions over the past 12 months. The second part provided details about the specific food items included within each food group identified in the first part. The validity and reproducibility of this questionnaire were satisfied in a dedicated study, including for fish consumption (15).

The data collected through the dietary questionnaire were used to estimate the average daily consumption of foods and beverages in grams per day. Fish consumption was assessed using the dietary questionnaire sent in 1993. In addition to analysing the overall fish consumption, we also examined the intake of lean fish (cod, whiting, hake, pollock, ling, dab, sole, haddock, coley) and fatty fish (salmon, trout, sardines, mackerel, and canned tuna), which specific consumptions were specified in the second part of the dietary questionnaire.

Daily nutrient intakes were obtained from the French food composition table provided by the French Information Centre on Food Quality (CIQUAL) (16). The daily nutritional intake of n-3 PUFA predominantly found in fish (EPA, DHA, and DPA) were considered in the analysis.

### 2.4 Estimation of POPs dietary intake

Food contamination levels were obtained from food contamination data performed by the French Agency for Food, Environmental, and Occupational Health and Safety (ANSES) during the second French Total Diet Study (17), the most comprehensive French food contamination database.

In summary, a total of 20,280 food items were acquired from eight French regions between 2007 and 2009. This led to the creation of 1,352 composite samples that were prepared “as consumed” (including processes such as peeling and frying) for the analysis of over 400 food chemical contaminants, as detailed by Sirot et al. (18).

In our study, we were focused on POPs. In particular, dietary intakes of non-dioxin-like PCBs (NDL-PCBs, continuous, ng/day), dioxins added to dioxin-like PCBs (dioxins + DL-PCBs continuous, TEQ, pg/day), and PBDEs (continuous, ng/day) were included in the analyses.

The estimation of participant’s dietary intake of those POPs was carried out by merging the E3N database containing food consumption levels and the ANSES database containing food contamination levels as detailed in the study of Mancini et al. (19). For each participant, the average daily dietary intake of each POP was obtained by summing, for each food item, the product of the average daily quantity consumed of that food item by the level of contamination of that food item with the POP of interest. Then, the dietary intake of each group of POPs (dioxins, DL-PCBs, NDL-PCBs, or PBDEs) was obtained by adding up the dietary intake of each congener. For this study, 17 dioxin congeners, six NDL-PCBs congeners, 12 DL-PCBs congeners, and eight PBDEs congeners were included in the analysis (Supplementary Table 1). To limit the overestimation of POPs intakes, we estimated those intakes according to the lower bound (LB) scenario, in which non-detected values were replaced by zero.

### 2.5 Assessment of other covariates

In order to respect temporality, covariates measured in several E3N questionnaires were selected in the second questionnaire (Q2) sent in 1992 to precede the dietary questionnaire (sent in 1993) which focused on the last 12 months dietary consumptions. We included physical activity measured at Q3 as it was not collected at Q2.

The adjustment variables were selected based on the literature and based on a directed acyclic graph (DAG; Supplementary Figure 1). The following covariates were included in the analyses: birth cohort (≤1930; (1930–1935]; (1935–1940]; (1940–1945]; >1945), education level (<12 years, 12 to 14 years, >14 years), smoking status at Q2 (never smoker; former smoker; current smoker), body mass index (BMI) at Q2 (continuous, kg/m^2^, derived from height and weight), menopausal status combined with recent use (within the past year) of menopausal hormone therapy (MHT) at Q2 (premenopausal; menopausal with recent MHT use; menopausal without recent MHT use; menopausal with missing data on MHT use), physical activity at Q3 (continuous, metabolic equivalents of task-hours/week (MET-h/week)), total energy intake excluding alcohol consumption at Q3 (continuous, kcal/day), alcohol consumption at Q3 (continuous, g of ethanol/day), red and processed meat consumption at Q3 (continuous, g/day), fruits and vegetables consumption at Q3 (continuous, g/day), and dairy products consumption at Q3 (continuous, g/day).

### 2.6 Study population

This study included all participants who had completed the dietary questionnaire sent in June 1993, totalling 74,522 women. For this analysis, we excluded participants with extreme energy intake to mitigate over-reporting or under-reporting (*n* = 1,491). In other words, participants with energy intake-to-requirements ratios in the bottom or top 1% of the distribution were excluded. Energy requirements were estimated using the basal metabolic rate (BMR) multiplied by the level of physical activity. To calculate BMR, we used the Schofield equation based on age, sex, and weight (20). Additionally, we excluded women who did not complete subsequent questionnaires after this dietary questionnaire (*n* = 446).

Our study population for all-cause mortality comprised 72,585 women. Subsequently, women with unknown causes of death (*n* = 169) were excluded, resulting in a study population of 72,416 women for analyses on cancer or cardiovascular disease mortality. Finally, for analyses on mortality by specific cancer type, women with unknown primary locations of cancer were also excluded (*n* = 178), resulting in a study population of 72,238 participants. The flow chart is presented in the Supplementary Figure 2.

### 2.7 Statistical analyses

#### 2.7.1 Descriptive analyses

The characteristics at baseline of the study population were described (mean and standard deviation for continuous variables, frequencies and proportions for categorical variables) for the total population and within each quartile group of fish consumption (in g/day). Spearman rank correlation tests between dietary exposure to POPs and fish consumption were also performed (Supplementary Table 2).

#### 2.7.2 Main analyses

Cox proportional hazard regression models, with age as the time scale, were employed to estimate Hazard Ratios (HR) and their 95% confidence intervals (CI). In the Cox models, fish consumption (in g/day), lean fish consumption (in g/day), fatty fish consumption (in g/day), and n-3 PUFA intake (sum of EPA, DPA, and DHA in g/day) were analysed separately as the primary exposure variables, treated as both a continuous and categorical variable, with the smallest quartile group serving as the reference. The outcomes of interest were the following: all-cause mortality, CVD mortality, cancer mortality, and mortality from specific types of cancer (breast, lung, and colorectal). To account for the role of total energy intake in the association between n-3 PUFA intake and the risk of mortality, we applied the energy adjustment residual method which consisted of substituting the n-3 PUFA intake main exposure variable with the residuals of the regression between n-3 PUFA intake and total energy intake (21).

Participants were followed from their age at Q3 response until the age at the first observed event, being the end of the follow-up (November 17, 2014), the date of death, or the date of completion of the last questionnaire, whichever occurred first. Furthermore, for analyses on specific causes of mortality, causes other than those of interest were censored at the date of death.

Five models were developed for each of the 4 main exposure variables (fish consumption, lean fish consumption, fatty fish consumption, and n-3 PUFA intake). Model 1 was unadjusted with age as the time scale. Model 2 was adjusted for the following covariates: birth cohort, education level, smoking status, BMI, menopausal status combined with recent use of MHT, physical activity, total energy intake excluding alcohol consumption, alcohol consumption, red and processed meat consumption, fruits and vegetables consumption, and dairy products consumption. We performed additional analyses further adjusting Model 2 for dietary intake of POPs: Model 3 was adjusted for dietary intake of dioxins added to DL-PCBs (TEQ, pg/day), Model 4 for dietary intake of NDL-PCBs (ng/day), and Model 5 for dietary intake of PBDEs (ng/day).

Restricted cubic spline (RCS) regression was used to assess dose–response relationships for all continuous variables and to examine deviations from linearity. If the linearity test was not respected (i.e., if the *p*-value of the non-linearity test was under 0.10), the continuous variable was considered non-linear, and RCS was used to model this variable. In these models, the number of knots for the main exposure variable was determined using the smallest Akaike information criterion (AIC) between three test models: 3 knots (at the 10^th^, 50^th^, and 90^th^ percentiles), 4 knots (at the 5^th^, 35^th^, 65^th^, and 95^th^ percentiles), and 5 knots (at the 5^th^, 27.5^th^, 50^th^, 72.5^th^, and 95^th^ percentiles). For continuous covariates, 4 knots (at the 5^th^, 35^th^, 65^th^, and 95^th^ percentiles) were used by default, as recommended by Harell (22). When the main exposure variable was linear, it was divided by its standard deviation to obtain estimates of the HRs for the increase of one standard deviation.

We imputed covariates with less than 5% of missing values with the median for continuous variables and with the modal category for categorical variables. We created an “unknown value” category when there were more than 5% missing values (only for menopausal status and recent MHT use).

A *p*-value less than 5% was considered statistically significant. We used SAS 9.4 software to build the database and R software version 4.1.0 to perform statistical analyses.

#### 2.7.3 Sensitivity analyses

In order to assess the impact of a potential reverse causation bias, we ran Model 2 after having included a 5-year exposure lag and excluded all cases and participants censored during the first 5 years of follow-up.

## 3 Results

### 3.1 General characteristics

The average follow-up was 19.0 years (standard deviation: 4.1). During the follow-up, 6,441 women died, including 896 due to CVD and 3,473 due to cancer (of which 953 due to breast cancer, 364 due to lung cancer, and 317 due to colorectal cancer).

Table 1 describes the baseline characteristics of the study population overall and according to quartile groups of fish consumption. The baseline characteristics of the study population according to quartile groups of n-3 PUFA are described in Supplementary Table 3.

**Table 1:** Baseline characteristics of the study population overall and among each quartile group of fish intake in the E3N cohort (*N* = 72,585).

		Fish intake (min-max, g/day)			
Characteristic^1^	All (0.0–302.6) *N* = 72,585	Q1 (0.0–17.3) *N* = 18,141	Q2 (17.4–28.8) *N* = 18,152	Q3 (28.9–44.5) *N* = 18,143	Q4 (44.6–302.6) *N* = 18,149
Vital status at the end of the follow-up					
Not death	66,144 (91.1)	16,423 (90.5)	16,589 (91.4)	16,658 (91.8)	16,474 (90.8)
Death	6,441 (8.9)	1718 (9.5)	1,563 (8.6)	1,485 (8.2)	1,675 (9.2)
Age (years)	52.9 (6.7)	52.9 (6.8)	52.6 (6.6)	52.8 (6.7)	53.4 (6.7)
Birth cohort					
≤ 1930	7,295 (10.1)	1931 (10.6)	1,677 (9.2)	1756 (9.7)	1931 (10.6)
(1930–1935]	9,996 (13.8)	2,494 (13.7)	2,333 (12.9)	2,443 (13.5)	2,726 (15.0)
(1935–1940]	14,710 (20.3)	3,559 (19.6)	3,562 (19.6)	3,669 (20.2)	3,920 (21.6)
(1940–1945]	17,811 (24.5)	4,393 (24.2)	4,486 (24.7)	4,484 (24.7)	4,448 (24.5)
> 1945	22,773 (31.4)	5,764 (34.8)	6,094 (33.6)	5,791 (31.9)	5,124 (28.2)
Education level					
< 12 years	8,190 (11.3)	2,399 (13.2)	2038 (11.2)	1906 (10.5)	1847 (10.2)
12 to 14 years	38,408 (52.9)	9,597 (52.9)	9,564 (52.7)	9,789 (54.0)	9,458 (52.1)
> 14 years	25,987 (35.8)	6,145 (33.9)	6,550 (36.1)	6,448 (35.5)	6,844 (37.7)
BMI (kg/m^2^)	22.7 (3.1)	22.5 (3.0)	22.6 (3.0)	22.7 (3.1)	23.2 (3.3)
≤ 18.5	2,645 (3.6)	846 (4.7)	691 (3.8)	595 (3.3)	513 (2.8)
(18.5–25]	56,794 (78.2)	14,414 (79.5)	14,485 (79.8)	14,215 (78.3)	13,680 (75.4)
> 25	13,146 (18.1)	2,881 (15.9)	2,976 (16.4)	3,333 (18.4)	3,956 (21.8)
Smoking status					
Never	40,286 (55.5)	10,305 (56.8)	10,121 (55.8)	10,055 (55.4)	9,805 (54.0)
Former	23,139 (31.9)	5,414 (29.8)	5,763 (31.7)	5,871 (32.4)	6,091 (33.6)
Current	9,160 (12.6)	2,422 (13.4)	2,268 (12.5)	2,217 (12.2)	2,253 (12.4)
Physical activity (MET-hours/week)	46.4 (43.4)	44.3 (44.3)	44.5 (41.5)	46.5 (41.4)	50.3 (45.9)
Menopausal status and recent use of MHT					
Premenopausal	37,313 (51.4)	9,345 (51.5)	9,808 (54.0)	9,458 (52.1)	8,702 (47.9)
Menopausal and recent MHT use	10,047 (13.8)	2,314 (12.8)	2,373 (13.1)	2,580 (14.2)	2,780 (15.3)
Menopausal and no recent MHT use	21,671 (29.9)	5,647 (31.1)	5,134 (28.3)	5,215 (28.7)	5,675 (31.3)
Menopausal and no information on whether and when MHT was used	3,554 (4.9)	835 (4.6)	837 (4.6)	890 (4.9)	992 (5.5)
Total Energy intake (kcal/day)	2210.6 (560.8)	2042.5 (528.5)	2186.3 (528.3)	2266.5 (551.9)	2346.9 (586.8)
Energy intake (excluding energy from alcohol, kcal/day)	2129.4 (544.3)	1970.7 (515.1)	2107.0 (514.1)	2182.8 (534.6)	2256.8 (569.9)
Alcohol consumption (g ethanol/day)	11.6 (13.9)	10.3 (13.4)	11.3 (13.5)	12.0 (13.8)	12.9 (14.8)
Fruits and vegetables consumption (g/day)	743.0 (298.5)	681.4 (297.0)	718.3 (284.3)	747.4 (284.1)	824.7 (309.3)
Red and processed meat consumption (g/day)	73.7 (39.5)	68.9 (40.3)	76.9 (39.2)	76.7 (39.3)	72.2 (38.5)
Dairy products consumption (g/day)	360.3 (210.0)	341.1 (206.2)	351.1 (203.2)	361.8 (204.3)	387.1 (222.9)
EPA + DHA + DPA dietary intake (mg/day)	501.5 (324.3)	212.2 (88.7)	368.1 (104.1)	527.1 (141.0)	898.4 (349.6)
EPA dietary intake (mg/day)	151.1 (108.5)	57.1 (27.8)	106.6 (34.6)	158.3 (48.4)	282.3 (121.4)
DPA dietary intake (mg/day)	65.5 (30.6)	42.5 (18.6)	57.0 (20.0)	68.8 (22.7)	93.6 (33.0)
DHA dietary intake (mg/day)	284.9 (191.2)	112.6 (49.5)	204.4 (57.5)	299.9 (78.5)	522.5 (203.7)
Non-dioxin-like PCBs dietary intake (ng/day)	151.9 (70.6)	95.9 (33.9)	127.3 (36.1)	155.5 (41.6)	228.8 (78.2)
Dioxins and dioxin-like PCBs dietary intake (TEQ, pg/day)	30.8 (12.1)	23.9 (9.6)	28.1 (9.6)	31.5 (9.9)	39.9 (12.7)
PBDEs dietary intake (ng/day)	42.0 (16.7)	32.5 (13.9)	38.6 (13.8)	43.3 (14.1)	53.8 (17.2)

1 *N* (%) for categorical variables; Mean (std) for continuous variables.

Overall, the average intake of fish was 34.0 g/day: 20.5 g/day of lean fish and 13.5 g/day of fatty fish. Regarding nutrient consumption, the average total intake of n-3 PUFA was 501.5 mg/day (standard deviation: 324.3), with a predominance of DHA (284.9 mg/day, standard deviation: 191.2), compared to EPA (151.1 mg/day, standard deviation: 108.5) and DPA (65.5 mg/day, standard deviation: 30.6).

Furthermore, a higher fish consumption (>44.6 g/day), compared to less than 17.3 g/day of fish consumption, corresponded to a higher dietary intake of POPs with an average intake of 228.8 vs. 95.9 ng/day for NDL-PCBs, 39.9 vs. 23.9 TEQ pg/day for dioxins and DL-PCBs, and 53.8 vs. 32.5 ng/day of PBDEs.

### 3.2 Fish consumption or n-3 PUFA intake and mortality

**Figure 1:**
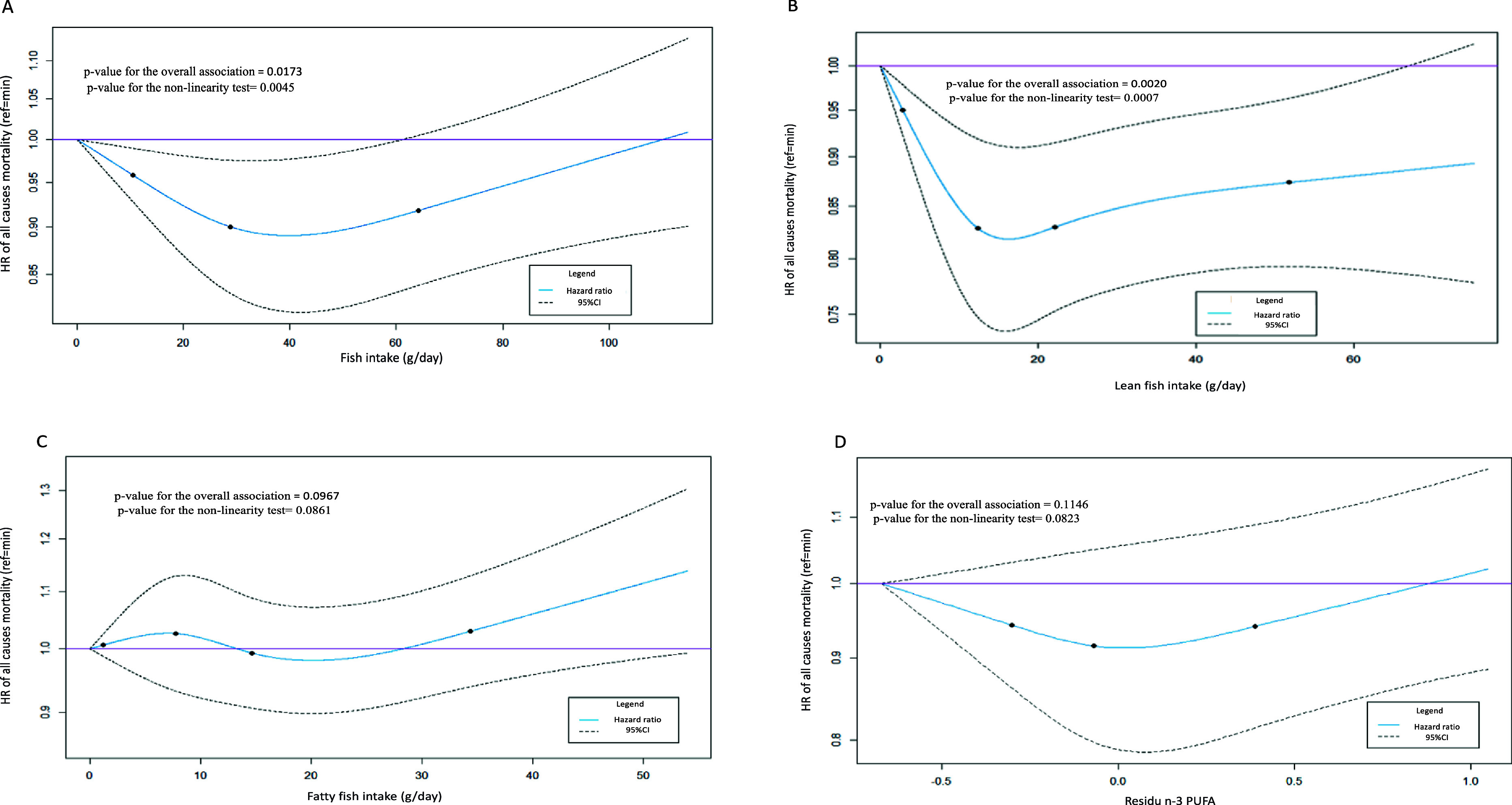
Restricted cubic splines of Model 2 of fish consumption **(A)**, lean fish consumption **(B)**, fatty fish consumption **(C)**, and n-3 PUFA intake residuals **(D)** in association with all-cause mortality risk in the E3N cohort (*N* = 72,585). The minimum value of the main exposure variable is taken as reference. Solid lines indicate HR and dashed lines indicate 95% CI. The points represent percentiles as follows: 3 knots at the 10^th^, 50^th^, and 90^th^ percentiles or 4 knots at the 5^th^, 35^th^, 65^th^, and 95^th^ percentiles.

In Model 2, the analysis revealed a U-shaped association between fish consumption and all-cause mortality (P overall association = 0.017; Table 2 and Figure 1A). The use of spline functions highlighted a reduction in the risk of all-cause mortality when fish consumption increased from 0 to 40 g/day, and then an inversion of the trend (Figure 1A). The association was also non-linear but not statistically significant between fish consumption and CVD mortality (P overall association = 0.281) and cancer mortality (P overall association = 0.179). The association between fish consumption and breast cancer mortality as well as colorectal cancer mortality was linear but not statistically significant (respectively, HR [CI95%]: 1.05 [0.98–1.13] and HR [CI95%]: 0.93 [0.81–1.06]). The analyses also revealed a statistically significant non-linear association between fish consumption and lung cancer mortality (P overall association = 0.005; Table 2 and Supplementary Figure 3).

**Table 2:** Hazard ratios (CI95%) estimated by Cox multivariable regression models for the association between fish consumption and mortality risk in the E3N cohort.

		*N* deaths	Model 1 HR (CI95%)	Model 2 HR (CI95%)
All-cause mortality (*N* = 72,585)	Fish intake (spline with 4 knots, g/day)	6,441		
	*p*-value for the overall association		<0.001	0.017
	*p*-value for the non-linearity test		<0.001	0.005
	Q1 (0.00–17.26)	1,718	1.00	1.00
	Q2 (17.27–28.77)	1,563	0.94 (0.88–1.01)	0.99 (0.91–1.05)
	Q3 (28.78–44.53)	1,485	0.87 (0.81–0.93)	0.92 (0.86–0.99)
	Q4 (44.54–302.57)	1,675	0.94 (0.88–1.01)	0.98 (0.92–1.05)
CVD mortality (*N* = 72,416)	Fish intake (spline with 3 knots, g/day)	896		
	*p*-value for the overall association		0.023	0.281
	*p*-value for the non-linearity test		0.006	0.088
	Q1 (0.00–17.26)	254	1.00	1.00
	Q2 (17.27–28.77)	204	0.85 (0.71–1.02)	0.89 (0.74–1.07)
	Q3 (28.78–44.53)	198	0.79 (0.66–0.95)	0.85 (0.70–1.03)
	Q4 (44.54–302.57)	240	0.90 (0.75–1.07)	0.95 (0.79–1.14)
Cancer mortality (*N* = 72,416)	Fish intake (spline with 3 knots, g/day)	3,473		
	*p*-value for the overall association		0.022	0.179
	*p*-value for the non-linearity test		0.006	0.065
	Q1 (0.00–17.26)	898	1.00	1.00
	Q2 (17.27–28.77)	873	0.99 (0.90–1.09)	1.01 (0.92–1.11)
	Q3 (28.78–44.53)	818	0.91 (0.83–1.01)	0.94 (0.85–1.04)
	Q4 (44.54–302.57)	884	0.96 (0.87–1.05)	0.97 (0.88–1.07)
Breast cancer mortality (*N* = 72,238)	Fish intake (linear term, g/day)	953	1.05 (0.98–1.13)	1.05 (0.98–1.13)
	*p*-value		0.144	0.150
	Q1 (0.00–17.26)	242	1.00	1.00
	Q2 (17.27–28.77)	243	1.01 (0.85–1.21)	1.05 (0.87–1.25)
	Q3 (28.78–44.53)	203	0.84 (0.70–1.01)	0.87 (0.72–1.06)
	Q4 (44.54–302.57)	265	1.08 (0.91–1.29)	1.11 (0.92–1.33)
Lung cancer mortality (*N* = 72,238)	Fish intake (spline with 3 knots, g/day)	364		
	*p*-value for the overall association		0.083	0.005
	*p*-value for the non-linearity test		0.092	0.002
	Q1 (0.00–17.26)	95	1.00	1.00
	Q2 (17.27–28.77)	116	1.24 (0.95–1.63)	1.29 (0.98–1.70)
	Q3 (28.78–44.53)	77	0.81 (0.60–1.10)	0.87 (0.64–1.18)
	Q4 (44.54–302.57)	76	0.78 (0.58–1.05)	0.86 (0.63–1.18)
Colorectal cancer mortality (*N* = 72,238)	Fish intake (linear term, g/day)	317	0.94 (0.82–1.07)	0.93 (0.81–1.06)
	*p*-value		0.319	0.258
	Q1 (0.00–17.26)	83	1.00	1.00
	Q2 (17.27–28.77)	74	0.92 (0.67–1.25)	0.90 (0.66–1.24)
	Q3 (28.78–44.53)	89	1.08 (0.80–1.45)	1.05 (0.78–1.42)
	Q4 (44.54–302.57)	71	0.83 (0.60–1.14)	0.81 (0.58–1.12)

Model 1: Adjustment on age (years) as time-scale. Model 2: M1 + birth cohort (≤1930, (1930–1935], (1935–1940], (1940–1945], >1945), education level (<12 years, 12 to 14 years, >14 years), smoking status (never smoker, former smoker, current smoker), physical activity (MET-hours/week), BMI (kg/m^2^), menopausal status and recent MHT use (premenopausal, menopausal and recent MHT use, menopausal and no recent MHT use, menopausal and no information on whether and when MHT was used), energy intake (excluding energy from alcohol, kcal/day), alcohol consumption (g ethanol/day), fruits and vegetables consumption (g/day), red and processed meat consumption (g/day), and dairy products consumption (g/day).

The analysis of the association between lean fish consumption and the risk of all-cause mortality in Model 2 revealed a statistically significant non-linear association (P overall association = 0.002), with a reduction of the risk when lean fish consumption increased up to 18 g/day after which a plateau was observed (Table 3 and Figure 1B). The associations between lean fish consumption and all other causes of death were not statistically significant (Table 3).

**Table 3:** Hazard ratios (CI95%) estimated by Cox multivariable regression models for the association between lean fish consumption and mortality risk in the E3N cohort.

		*N* deaths	Model 1 HR (CI95%)	Model 2 HR (CI95%)
All-cause mortality (*N* = 72,585)	Lean fish intake (spline with 4 knots, g/day)	6,441		
	*p*-value for the overall association		<0.001	0.002
	*p*-value for the non-linearity test		<0.001	0.001
	Q1 (0.00–9.32)	1,729	1.00	1.00
	Q2 (9.33–17.19)	1,482	0.87 (0.82–0.93)	0.91 (0.85–0.98)
	Q3 (17.20–26.94)	1,602	0.87 (0.82–0.93)	0.93 (0.86–0.99)
	Q4 (26.94–206.00)	1,628	0.89 (0.83–0.95)	0.93 (0.87–1.00)
CVD mortality (*N* = 72,416)	Lean fish intake (spline with 4 knots, g/day)	896		
	*p*-value for the overall association		0.094	0.287
	*p*-value for the non-linearity test		0.043	0.117
	Q1 (0.00–9.32)	251	1.00	1.00
	Q2 (9.33–17.19)	200	0.83 (0.69–1.00)	0.89 (0.74–1.08)
	Q3 (17.20–26.94)	213	0.79 (0.66–0.95)	0.85 (0.71–1.03)
	Q4 (26.94–206.00)	232	0.84 (0.70–1.00)	0.90 (0.75–1.08)
Cancer mortality (*N* = 72,416)	Lean fish intake (spline with 4 knots, g/day)	3,473		
	*p*-value for the overall association		0.025	0.126
	*p*-value for the non-linearity test		0.011	0.049
	Q1 (0.00–9.32)	905	1.00	1.00
	Q2 (9.33–17.19)	838	0.93 (0.85–1.02)	0.96 (0.84–1.05)
	Q3 (17.20–26.94)	898	0.94 (0.86–1.03)	0.98 (0.89–1.07)
	Q4 (26.94–206.00)	832	0.89 (0.81–0.98)	0.92 (0.83–1.01)
Breast cancer mortality (*N* = 72,238)	Lean fish intake (linear term, g/day)	953	1.02 (0.95–1.09)	1.02 (0.95–1.09)
	*p*-value		0.544	0.595
	Q1 (0.00–9.32)	236	1.00	1.00
	Q2 (9.33–17.19)	235	0.99 (0.82–1.18)	1.02 (0.85–1.22)
	Q3 (17.20–26.94)	241	0.97 (0.81–1.16)	1.01 (0.84–1.21)
	Q4 (26.94–206.00)	241	1.01 (0.85–1.21)	1.04 (0.86–1.24)
Lung cancer mortality (*N* = 72,238)	Lean fish intake (spline with 4 knots, g/day)	364		
	*p*-value for the overall association		0.002	0.058
	*p*-value for the non-linearity test		0.001	0.026
	Q1 (0.00–9.32)	113	1.00	1.00
	Q2 (9.33–17.19)	92	0.82 (0.62–1.08)	0.87 (0.66–1.15)
	Q3 (17.20–26.94)	87	0.73 (0.55–0.97)	0.81 (0.61–1.07)
	Q4 (26.94–206.00)	72	0.62 (0.46–0.83)	0.70 (0.51–0.94)
Colorectal cancer mortality (*N* = 72,238)	Lean fish intake (linear term, g/day)	317	0.93 (0.82–1.05)	0.93 (0.81–1.05)
	*p*-value		0.245	0.244
	Q1 (0.00–9.32)	89	1.00	1.00
	Q2 (9.33–17.19)	76	0.86 (0.64–1.17)	0.85 (0.63–1.16)
	Q3 (17.20–26.94)	78	0.83 (0.61–1.12)	0.82 (0.60–1.11)
	Q4 (26.94–206.00)	74	0.80 (0.59–1.09)	0.78 (0.57–1.07)

Model 1: Adjustment on age (years) as time-scale. Model 2: M1 + birth cohort (≤1930, (1930–1935], (1935–1940], (1940–1945], >1945), education level (<12 years, 12 to 14 years, >14 years), smoking status (never smoker, former smoker, current smoker), physical activity (MET-hours/week), BMI (kg/m^2^), menopausal status and recent MHT use (premenopausal, menopausal and recent MHT use, menopausal and no recent MHT use, menopausal and no information on whether and when MHT was used), energy intake (excluding energy from alcohol, kcal/day), alcohol consumption (g ethanol/day), fruits and vegetables consumption (g/day), red and processed meat consumption (g/day), and dairy products consumption (g/day).

For fatty fish consumption, the association with all-cause mortality was non-linear although not statistically significant (P overall association = 0.097; Table 4 and Figure 1C). A non-linear and statistically significant association was identified for fatty fish consumption in relation to cancer mortality (P overall association = 0.035), while a positive, linear and statistically significant association was observed between fatty fish consumption and breast cancer mortality (HR [CI95%]: 1.07 [1.01–1.15]). The associations between fatty fish consumption and all other causes of death were not statistically significant (Table 4).

**Table 4:** Hazard ratios (CI95%) estimated by Cox multivariable regression models for the association between fatty fish consumption and mortality risk in the E3N cohort.

		*N* deaths	Model 1 HR (CI95%)	Model 2 HR (CI95%)
All-cause mortality (*N* = 72,585)	Fatty fish intake (spline with 4 knots, g/day)	6,441		
	*p*-value for the overall association		<0.001	0.097
	*p*-value for the non-linearity test		<0.001	0.086
	Q1 (0.00–5.91)	1,673	1.00	1.00
	Q2 (5.92–10.88)	1,651	1.03 (0.96–1.10)	1.06 (0.99–1.14)
	Q3 (10.89–18.13)	1,542	0.97 (0.90–1.04)	1.01 (0.94–1.09)
	Q4 (18.14–186.57)	1,575	0.98 (0.92–1.05)	1.02 (0.95–1.10)
CVD mortality (*N* = 72,416)	Fatty fish intake (spline with 4 knots, g/day)	896		
	*p*-value for the overall association		0.303	0.853
	*p*-value for the non-linearity test		0.173	0.858
	Q1 (0.00–5.91)	236	1.00	1.00
	Q2 (5.92–10.88)	230	1.05 (0.87–1.25)	1.10 (0.91–1.32)
	Q3 (10.89–18.13)	215	0.99 (0.82–1.19)	1.07 (0.88–1.29)
	Q4 (18.14–186.57)	215	0.98 (0.81–1.18)	1.05 (0.86–1.27)
Cancer mortality (*N* = 72,416)	Fatty fish intake (spline with 4 knots, g/day)	3,473		
	*p*-value for the overall association		0.017	0.035
	*p*-value for the non-linearity test		0.017	0.030
	Q1 (0.00–5.91)	854	1.00	1.00
	Q2 (5.92–10.88)	921	1.10 (1.00–1.21)	1.06 (0.99–1.14)
	Q3 (10.89–18.13)	845	1.01 (0.92–1.11)	1.00 (0.93–1.07)
	Q4 (18.14–186.57)	853	1.02 (0.93–1.12)	1.01 (0.94–1.08)
Breast cancer mortality (*N* = 72,238)	Fatty fish intake (linear term, g/day)	953	1.07 (1.01–1.14)	1.07 (1.01–1.15)
	*p*-value		0.030	0.038
	Q1 (0.00–5.91)	224	1.00	1.00
	Q2 (5.92–10.88)	247	1.11 (0.93–1.33)	1.14 (0.95–1.36)
	Q3 (10.89–18.13)	228	1.03 (0.86–1.24)	1.06 (0.89–1.27)
	Q4 (18.14–186.57)	254	1.15 (0.96–1.38)	1.17 (0.98–1.41)
Lung cancer mortality (*N* = 72,238)	Fatty fish intake (spline with 4 knots, g/day)	364		
	*p*-value for the overall association		0.387	0.771
	*p*-value for the non-linearity test		0.257	0.471
	Q1 (0.00–5.91)	91	1.00	1.00
	Q2 (5.92–10.88)	108	1.21 (0.91–1.60)	1.24 (0.94–1.64)
	Q3 (10.89–18.13)	84	0.94 (0.70–1.27)	0.98 (0.73–1.33)
	Q4 (18.14–186.57)	81	0.91 (0.67–1.22)	0.99 (0.72–1.34)
Colorectal cancer mortality (*N* = 72,238)	Fatty fish intake (linear term, g/day)	317	0.97 (0.86–1.10)	0.95 (0.84–1.08)
	*p*-value		0.643	0.454
	Q1 (0.00–5.91)	83	1.00	1.00
	Q2 (5.92–10.88)	76	0.94 (0.69–1.28)	0.92 (0.67–1.26)
	Q3 (10.89–18.13)	84	1.04 (0.77–1.41)	1.01 (0.74–1.37)
	Q4 (18.14–186.57)	74	0.92 (0.67–1.25)	0.87 (0.63–1.21)

Model 1: Adjustment on age (years) as time-scale. Model 2: M1 + birth cohort (≤1930, (1930–1935], (1935–1940], (1940–1945], >1945), education level (<12 years, 12 to 14 years, >14 years), smoking status (never smoker, former smoker, current smoker), physical activity (MET-hours/week), BMI (kg/m^2^), menopausal status and recent MHT use (premenopausal, menopausal and recent MHT use, menopausal and no recent MHT use, menopausal and no information on whether and when MHT was used), energy intake (excluding energy from alcohol, kcal/day), alcohol consumption (g ethanol/day), fruits and vegetables consumption (g/day), red and processed meat consumption (g/day), and dairy products consumption (g/day).

The analysis of the association between n-3 PUFA intake residuals and the risk of all-cause mortality in Model 2 showed a non-linear and not statistically significant association (P overall association = 0.115; Table 5 and Figure 1D). A statistically significant, positive and linear association was revealed between n-3 PUFA intake residuals and the risk of breast cancer mortality (HR [CI95%]: 1.08 [1.01–1.15]). The associations between n-3 PUFA intake residuals and all other causes of death were not statistically significant (Table 5).

**Table 5:** Hazard ratios (CI95%) estimated by Cox multivariable regression models for the association between n-3 PUFA intake residuals and mortality risk in the E3N cohort.

		*N* deaths	Model 1 HR (CI95%)	Model 2 HR (CI95%)
All-cause mortality (*N* = 72,585)	n-3 PUFA intake residuals (spline with 3 knots)	6,441		
	*p*-value for the overall association		0.003	0.115
	*p*-value for the non-linearity test		0.003	0.082
	Q1 (−0.67—0.21)	1,660	1.00	1.00
	Q2 (−0.22—0.07)	1,540	0.95 (0.88–1.01)	0.96 (0.89–1.03)
	Q3 (−0.08–0.13)	1,571	0.97 (0.90–1.04)	0.99 (0.92–1.06)
	Q4 (0.14–4.15)	1,670	0.99 (0.92–1.05)	1.00 (0.93–1.07)
CVD mortality (*N* = 72,416)	n-3 PUFA intake residuals (spline with 3 knots)	896		
	*p*-value for the overall association		0.712	0.892
	*p*-value for the non-linearity test	219	0.654	0.941
	Q1 (−0.67—0.21)	223	1.00	1.00
	Q2 (−0.22—0.07)	217	1.05 (0.87–1.27)	1.06 (0.88–1.29)
	Q3 (−0.08–0.13)	237	1.03 (0.85–1.24)	1.06 (0.88–1.28)
	Q4 (0.14–4.15)		1.05 (0.87–1.26)	1.07 (0.88–1.29)
Cancer mortality (*N* = 72,416)	n-3 PUFA intake residuals (spline with 3 knots)	3,473		
	*p*-value for the overall association		0.016	0.072
	*p*-value for the non-linearity test		0.027	0.060
	Q1 (−0.67—0.21)	884	1.00	1.00
	Q2 (−0.22—0.07)	835	0.95 (0.87–1.05)	0.95 (0.86–1.05)
	Q3 (−0.08–0.13)	862	0.98 (0.90–1.08)	0.98 (0.89–1.08)
	Q4 (0.14–4.15)	892	0.99 (0.90–1.09)	0.98 (0.89–1.07)
Breast cancer mortality (*N* = 72,238)	n-3 PUFA intake residuals (linear term)	953	1.08 (1.01–1.15)	1.08 (1.01–1.15)
	*p*-value		0.019	0.027
	Q1 (−0.67—0.21)	227	1.00	1.00
	Q2 (−0.22—0.07)	232	1.03 (0.86–1.23)	1.05 (0.87–1.26)
	Q3 (−0.08–0.13)	236	1.05 (0.87–1.26)	1.08 (0.90–1.30)
	Q4 (0.14–4.15)	258	1.13 (0.95–1.35)	1.14 (0.95–1.37)
Lung cancer mortality (*N* = 72,238)	n-3 PUFA intake residuals (spline with 3 knots)	364		
	*p*-value for the overall association		0.235	0.062
	*p*-value for the non-linearity test		0.091	0.500
	Q1 (−0.67—0.21)	99	1.00	1.00
	Q2 (−0.22—0.07)	87	0.89 (0.66–1.18)	0.86 (0.64–1.14)
	Q3 (−0.08–0.13)	93	0.95 (0.71–1.26)	0.92 (0.69–1.22)
	Q4 (0.14–4.15)	85	0.84 (0.63–1.13)	0.85 (0.64–1.14)
Colorectal cancer mortality (*N* = 72,238)	n-3 PUFA intake residuals (linear term)	317	0.96 (0.85–1.09)	0.95 (0.84–1.08)
	*p*-value		0.514	0.439
	Q1 (−0.67—0.21)	89	1.00	1.00
	Q2 (−0.22—0.07)	73	0.83 (0.61–1.13)	0.83 (0.61–1.13)
	Q3 (−0.08–0.13)	76	0.86 (0.64–1.17)	0.86 (0.63–1.17)
	Q4 (0.14–4.15)	79	0.87 (0.64–1.17)	0.86 (0.63–1.16)

Model 1: Adjustment on age (years) as time-scale. Model 2: M1 + birth cohort (≤1930, (1930–1935], (1935–1940], (1940–1945], >1945), education level (<12 years, 12 to 14 years, >14 years), smoking status (never smoker, former smoker, current smoker), physical activity (MET-hours/week), BMI (kg/m^2^), menopausal status and recent MHT use (premenopausal, menopausal and recent MHT use, menopausal and no recent MHT use, menopausal and no information on whether and when MHT was used), energy intake (excluding energy from alcohol, kcal/day), alcohol consumption (g ethanol/day), fruits and vegetables consumption (g/day), red and processed meat consumption (g/day), and dairy products consumption (g/day).

Results remained virtually unchanged for the four exposure variables in relation to all outcomes tested when further adjusting Model 2 for dietary intake of dioxins and DL-PCBs (Model 3), NDL-PCBs (Model 4), or PBDEs (Model 5; Supplementary Tables 4–7).

### 3.3 Sensitivity analyses

Similar results to those of the main analyses were observed for the association between fish consumption and all-cause mortality after having included a 5-year exposure lag and excluded 1,663 participants who died or were censored during the first 5 years of follow-up (data not shown).

## 4 Discussion

This study allowed us to identify a non-linear (U-shaped) association between fish consumption and the risk of all-cause mortality. Indeed, the association between fish consumption and all-cause mortality was inverse up to consumption of 40 g/day: at this consumption level, the risk of all-cause mortality was reduced by around 10% compared to women whom did not consume fish. After this threshold, an inversion of the trend was observed. A similar but stronger association was observed when considering lean fish consumption, while the association between fatty fish consumption or n-3 PUFA intake with all-cause mortality risk was always non-linear but did not reach statistical significance. Interestingly, when observing results for cause-specific mortality risk, a non-linear association between fish consumption was observed in relation to lung cancer mortality, while a positive and linear association was observed between fatty fish consumption or n-3 PUFA intake with respect to breast cancer mortality.

In line with our finding, a meta-analysis similarly identified a U-shaped association between fish consumption and the mortality risk in studies conducted within Western countries (7). To explain the U-shape observed in the association between fish consumption and the risk of all-cause mortality, it had been hypothesised that dietary exposure to POPs could attenuate the beneficial health effects of fish consumption. Indeed, in some studies, the complex interaction between POPs and diet was reported, for example highlighting that POPs have the potential to disrupt the metabolism of beneficial nutrients (23–25). Nevertheless, the results obtained in our study do not allow us to confirm this hypothesis since our results remain virtually unchanged after adjusting the models for dioxins and DL-PCBs, NDL-PCBs, or PBDEs dietary intake. On the other hand, based on this study, no conclusion concerning the role played by other possible contaminants present in fish can be driven. Indeed, the form of association observed in our study could still be explained by the presence of other contaminants such as heavy metals (mercury, cadmium), polycyclic aromatic hydrocarbons (mainly in smoked fish), organochlorine pesticides, and per-and poly fluor alkylated substances.

Moreover, divergent results have been observed among studies conducted in different geographical regions. Several Asian studies have reported an inverse and linear association between fish consumption and all-cause mortality (6, 8, 26, 27). This discrepancy could be explained by geographical variations of n-3 PUFA and POPs in fish (28, 29). It could also be explained by differences in fish preparation methods between the populations studied. Depending on the region, people in Asian countries tend to eat fish either raw, steamed, or sauteed, whereas frying may be more common in Europe (30). The study by Gadiraju et al. shows that frying food causes oxidative degradation, leading to the formation of oxidised compounds and, consequently, an alteration in nutritional properties (31). In addition, a prospective Australian study showed that consumption of non-fried fish was inversely associated with the risk of CVD mortality in women (HR [CI95%]: 0.64 [0.45–0.91]), whereas total fish consumption was not (32). These data suggest that the method of cooking fish, particularly deep-fried fish, could have an impact on the beneficial effects of fish consumption, making it an important subject for future research aimed at elucidating the potential factors that explain these differences.

Concerning cause-specific mortality risk, in agreement with our results, an inverse association between fish consumption and lung cancer incidence has been reported in a meta-analysis including 20 studies (33). On the other hand, the relationship between fish consumption and breast cancer incidence has not yet been fully elucidated and conflicting results have been observed, with some studies suggesting that high consumption of fatty fish is associated with a reduced risk for breast cancer (34, 35), while others observing a positive association between fatty fish consumption and breast cancer (36). Finally, in contrast to many previous studies (37–39), our analyses did not highlight any statistically significant effect on CVD mortality neither due to fish consumption (nor lean, nor fatty) nor to n-3 PUFA intake.

### 4.1 Strengths and limitations

Several limitations need to be considered when interpreting the results of the present study. Firstly, the generalisability of our results should be treated with caution. Indeed, the E3N cohort is composed of middle-aged French women working for the French national education system, which are leaner and with a higher education level (40), therefore not representative of the French general population. Moreover, concerning the comparison of fish consumption across European countries, some authors concluded that substantial variations exist (41). In addition, food intake is assessed using a self-administered questionnaire, which is subject to misestimation. It is recognised that dietary questionnaires can be affected by social desirability bias and memory bias due to difficulties in recalling food consumption over the last 12 months. These biases can lead to errors in the measurement of fish consumption, n-3 PUFA intake, and exposure to POPs. Nevertheless, the exclusion of energy outliers allows us to take account of under-and over-reporting. Moreover, due to lack of information, we did not consider fish cooking methods in our study, which might have impacted the results. In addition, the questionnaire was completed at baseline, assuming that dietary habits remained unchanged over time. Variations in dietary habits could increase exposure misclassification. However, we assume that the diets of middle-aged women varied little over time as suggested by Thorpe et al. (42). Furthermore, even though we have many adjustment variables, we cannot exclude the presence of residual and unmeasured confounding in the estimation of our associations. Finally, we cannot exclude that the fish contamination levels with POPs have varied between the time when the food consumption data were collected in the E3N cohort (1993) and when the food contamination levels were assessed (2007 and 2009), leading to a possible misclassification of POPs dietary intakes. However, as POPs are persistent in the environment, their concentration in food items varies slowly overtime. Moreover, even if there is a decrease in food items in the time elapsed, POPs are ubiquitous in the environment, so we can assume that this decrease is comparable in all food items, leading to a good classification of participants between them.

Nevertheless, this study presents several strengths. To our knowledge, this is the first epidemiological study to analyse the association between fish consumption and mortality risk, taking into account the dietary exposure to several POPs, highly present in fish. Moreover, to better characterise the effect of fish consumption on health, in the present study it was also possible to make the distinction in the consumption of lean and fatty fish, identified according to lipid content, and to explore the effect of n-3 PUFA intake. In addition, thanks to the long follow-up available in the E3N cohort and the large number of participants, it was possible to investigate the long-term effects of fish consumption and n-n-3 PUFA intake in relation to mortality while ensuring high statistical power. Furthermore, the availability of extensive data on causes of death enabled us to study associations between fish consumption and n-3 PUFA intake, and different specific causes of mortality. Finally, the study population has a low rate of loss to follow-up and a low proportion of missing data, reflecting the quality of the data available in the E3N cohort allowing to adjust for many potential confounding factors.

## 5 Conclusion

Our study is the first to evaluate the impact of fish consumption, making a distinction between lean and fatty fish, and of n-3 PUFA intake on the risk of all-cause and cause-specific mortality while considering the potential role of dietary exposure to several chemical contaminants. The results highlight a U-shaped association between fish consumption and the risk of all-cause mortality and suggest that consumption of lean fish is the main driver of this association. Further studies are needed to better clarify this non-linear association and the differences between lean and fatty fish in order to provide scientific evidence necessary to develop precise dietary recommendations to the general population.

## Data Availability

The data analyzed in this study is subject to the following licenses/restrictions: sensitive data, available upon request. Requests to access these datasets should be directed to francesca.mancini@gustaveroussy.fr.
